# Editor’s introduction to this issue (G&I 19:4, 2021)

**DOI:** 10.5808/gi.19.4.e1

**Published:** 2021-12-31

**Authors:** Taesung Park

**Affiliations:** Department of Statistics, Seoul National University, Seoul 08826, Korea

This issue contains one review article, 12 original articles, one application note, and two opinions. In this editorial, I would like to focus on three articles about genetic association studies. The first article, by Wonil Chung (Soongsil University, Korea), provides a good review of statistical models and computational tools for predicting complex traits and diseases. The author focused on utilizing large-sample public biobanks to perform accurate polygenic predictions based on genetic variants across the whole genome based on polygenic risk scores (PRS). The authors reviewed various statistical methodologies and diverse computational tools that have been developed to estimate PRS more accurately from genome-wide association studies (GWASs). The author emphasized that the successful utilization of PRS tools requires a thorough understanding of what the underlying model is and how to specify the parameters to achieve the best performance. I think that this review is quite informative for researchers working on PRS computation.

Next, an original article by Young Jin Kim’s group at National Institute of Health, Korea also presents a large-scale GWAS. The Division of Genome Science, Department of Precision Medicine, National Institute of Health of Korea, with which the authors are affiliated, has been in charge of the Korean Genome Epidemiology Study (KoGES) since 2001 [1]. KoGES is a cohort study focusing on the prevention of major chronic diseases such as type 2 diabetes (T2D) and hypertension. The Korea Biobank Array (KBA) was recently developed for genomic studies in the Korean population. The optimized KBA comprises approximately 830K variants [2]. Using 125,850 samples from a Korean population genotyped by the KBA, the authors validated known associations with T2D and related metabolic traits. To the best of my knowledge, this is one of the largest GWASs for T2D in Korean population. The authors considered the imputed datasets available in the KBA genomic data, publicly available East-Asian T2D summary statistics, and the linkage disequilibrium among the variants. The authors identified 1,837 statistically significant (p < 0.05) variants. Although the 0.05 threshold is relatively non-stringent, the identified variants can be used for valuable scientific evidence in future study designs, evaluations of the current power of GWASs, and future applications in precision medicine for the Korean population.

Next, an original article by Jong-Keuk Lee’s group at the Asan Institute for Life Sciences, Asan Medical Center, Seoul, Korea presents a genetic study identifying rare coding variants associated with Kawasaki disease (KD) by whole-exome sequencing. Although previous GWASs have successfully identified common variants associated with KD, this study is the first to focus on rare variants of KD in the Korean population. The authors identified five rare coding variants associated with KD, which I think will provide insights into new candidate genes and genetic variants potentially involved in the development of KD.

So far, I have provided comments on three articles about genetic association studies. The first review article provided the most up-to-date review on PRS. The second article presented novel variants for T2D from the largest GWAS in Korea. The third article summarized rare variants for KD identified by whole-exome sequencing. I think that both common variants and rare variants are informative for a disease. In addition to the identification of variants relevant to the disease of interest, it is also important to make accurate predictions based on these genetic variants for the prevention or personalized treatment of a disease (e.g., T2D or KD). I hope that the variants reported in these two original articles can be further utilized to develop PRS for these diseases in the Korean population.
